# Death of Eminent Physicians

**Published:** 1853-03

**Authors:** 


### DEATHS OF EMINENT PHYSICIANS.

Within a short time past several of the most noted members of the medical profession in Europe have died. The death of Pereira, the author of the great work on Materia Medica, was announced several weeks since. Dr. George Gregory, the eminent author and physician, died a few days after him; and we have just seen that the great chemist, Orfila, has been called away from the field of his labors. All these names are familiar to medical readers in every part of the world. From a somewhat extended biographical notice of Dr. Pereira in the London Lancet, we copy the following:

The subject of our present memoir was born in the parish of Shoreditch, London, on the 22d of May, 1804. He received the rudiments of his education at some of the small schools in the neighborhood; and when he was about ten years of age he was placed under the tuition of a classical master of no ordinary attainments. This gentleman kept an academy at No. 10 Queen street, Finsbury, and Pereira remained with him for a period of four years, during which time he obtained the friendship of his preceptor, who was accustomed to speak of him as a boyo f considerable merit. When he left school he manifested a strong desire to enter the medical profession; and accordingly, at the age of fifteen, or a little earlier, he was articled as an apprentice to Mr. Latham, an apothecary of the City-road. There he remained between two and three years, when his master became the subject of mental disease. This led to the breaking up of the practice and to the cancelling of Pereira's indentures. While he was with Mr. Latham he studied hard at his classics, and he drew a vocabulary of Latin terms for his guidance in dispensing.

In 1842 he gave two short courses of lectures at the rooms of the Pharmaceutical Society, and, in the year following, he was appointed its first professor. During that year he published his work on Food and Diet, and he was elected on the Council of the Royal Society. By that time, his practice as a physician had become rather extensive, and as it was rapidly increasing, he determined to throw aside his more scientific pursuits. Accordingly, in 1844, he resigned a part of the course of chemistry at the London Hospital into the hands of Dr. Letheby; in 1845 he gave up a larger portion of it; and in 1846 he relinquished it altogether. He continued, however, to lecture on Materia Medica at both the Hospital and the Pharmaceutical Society, and there is no reason for believing that he contemplated any change in this matter until the new regulations of the Apothecaries' Society transferred his course to the summer session. This arrangement intefered with his usual habits, and also with his ideas of the importance of the subject, and consequently, in 1850, he resigned his lectureship at the Hospital, though he still con tinued to deliver a winter course at the Pharmaceutical Society. In 1845 he was elected a fellow of the College, and, in 1851, he became a full physician at the Hospital. He had now reached the summit of his ambition: his reputation as an author was established, and the rewards of his industry was falling thick about him. He was a fellow of many scientific societies, he was in constant communication with the learned of all countries; he was intimately connected with many of the greatest institutions of the metropolis, and was, in fact, their brightest ornament; he had collected around him a large circle of friends and admirers, and he saw before him the prospect of wealth and happiness. In the midst of all this, however, he was stricken down, and that so suddenly, that he had hardly time to take leave of those who were about him. He died on the 20th of January last, and within one week of health and hope he was placed in his last resting place. His funeral took place at Kensal-green, on the Thursday following, in the presence of a large number of friends and pupils, who mourned in silent grief.

A retrospect of the labors of this distinguished physician will show that he was a man of no ordinary capacity, that he was characterized by qualities which always ensure success. He had an unquenchable thirst for knowledge, an indefatigable spirit, unbounded industry, and a determination of purpose that was irresistible. Whatsoever he did he did well, and he therefore made his performances as valuable to others as they were creditable to himself. This is evidenced by the works to which we have alluded, for they have made for him the reputation which he possessed, and have given to others the means of acquiring knowledge which is not to be found elsewhere. The great peculiarity of his efforts is, that he aimed more at bringing within our reach the treasures of other men's minds, than of exposing those of his own. He has, indeed, been charged with a want of originality, and, most certainly, if we estimate him by the value of his own independent researches, he is open to such a charge; but it must also be admitted that it is an equally useful element of the human mind, that faculty which urges men to gather up the scattered facts of science, and to mould them into a shape that may be made available to all. This has our author accomplished, and the result of his labors will endure beyond the efforts of originality, for they will last hrough all time, as a monument of his industry.

